# pH and Pb co-regulate soil bacterial communities and C/N/S Cycling processes in valley-type landfills under multi-factor interactions

**DOI:** 10.3389/fmicb.2026.1780940

**Published:** 2026-03-24

**Authors:** Guangli Xiao, Yi Wu, Junlun Meng, Xiaoling Wu, Qili Gou, Xi Han, Minghui Qi, Yuqing Zhang, Xiaoxun Xu, Shirong Zhang

**Affiliations:** 1 College of Environmental Sciences, Sichuan Agricultural University, Chengdu, China; 2 Sichuan Institute of Energetical and Geological Survey, Chengdu, China; 3 Key Laboratory of Investigation and Monitoring, Protection and Utilization for Cultivated Land Resources, Ministry of Natural Resources, Chengdu, China; 4 Sichuan Academy of Eco-Environmental Sciences, Sichuan Province Engineering Technology Research Center of Emerging Contaminants Treatment and Environmental Health, Chengdu, China

**Keywords:** C/N/S cycle, co-occurrence network, functional prediction, landfill leachate, soil bacterial community

## Abstract

Valley-type landfills, a globally widespread source of contamination, produce leachate that significantly affecting adjacent soils. Clarifying leachate’s contribution to global biogeochemical cycles requires investigating soil bacterial communities related to C/N/S cycling, but few studies have explored interactive effects of multiple factors (season, soil layer, distance). To fill this gap, 144 soil samples were collected across three hydrological seasons (dry, normal, wet) from a representative valley-type landfill in southern Sichuan, China. Results showed that leachate exposure increased the relative abundances of dominant phyla such as Proteobacteria (by over 200%) and Acidobacteriota (by over 30%), as well as Acidobacteriota’s genus Pseudolabrys, while reducing the relative abundance of the sensitive phylum Chloroflexi by 50%. During normal and wet seasons, bacterial diversity at leachate-affected sites was up to twice that of clean soil, with more complex co-occurrence networks. Redundancy analysis and partial least squares path modeling (PLS-PM) revealed that soil pH (positive) and Pb (negative) were key factors regulating bacterial communities and C/N/S cycling functions. Distance emerged as the dominant predictor of bacterial community and function in random forest analyses. This work elucidates the response of C/N/S cycling-related bacterial communities and functions to leachate under multi-factor interactions, providing a scientific basis for understanding valley-type landfills’ role in global biogeochemical cycles.

## 1 Introduction

Landfilling is the most common method for solid waste treatment (Oliveira et al., 2024; Wei et al., 2024). The number of landfills is increasing dramatically in both developed countries (e.g., the United States) and most developing countries (Naeem et al., 2025; Ochs et al., 2024). Landfill leachate contains various pollutants, including heavy metals, inorganic salts, and organic matter (Igwegbe et al., 2024; Zhou et al., 2025). The acidity of leachate varies significantly depending on the fermentation stage of the landfill, with pH values ranging from 4 to 9 (Renou et al., 2008; Lepota et al., 2025; Kjeldsen et al., 2002), resulting in different levels of environmental hazards. Due to poor management, inadequate liner systems, insufficient landfill capacity, or natural disasters, leachate leakage can contaminate the surrounding soil and ultimately threaten ecosystems (Sha et al., 2023; Teng et al., 2021).

Traditional landfill design aims to maximize solid waste accommodation and at low cost, making valley terrain a common choice for sites (Cai et al., 2024; Cheng et al., 2024; Zakaria et al., 2024). Landfills built in these areas without professional design pose risks to the surrounding environment (Oliveira et al., 2024; Saha et al., 2023). Numerous investigations have emphasized the safety of valley-type landfill site selection and hydrogeological characteristics, as well as their impacts on groundwater pollution (Arabeyyat et al., 2024; da Luz et al., 2025). Landfill leachate affects the structure and function of bacterial communities in the surrounding soil (He R. et al., 2023; Hui et al., 2025). The critical biotic and abiotic controlling factors in this process, together with the effects of leachate leakage on soil microbial communities in valley-type landfills, have not been fully investigated. Resolving these questions is essential for characterizing the inherent features of valley-type landfills as a distinct ecological system. Landfills is widely recognized as one of the most anthropogenically impacted ecosystems. The valley-type geomorphology significantly increases the complexity of leachate migration dynamics and contaminant transport behavior.

Seasonal variations, especially in precipitation, drive the leaching and migration of leachate pollutants, thereby altering heterogeneity of soil microenvironment the in leachate-affected areas. These changes affect soil physicochemical properties and may directly impact soil bacterial structure and function (Marciulynas et al., 2022; Yin et al., 2022). Previous studies have shown that leachate pollutants migration is enhanced during the wet season, posing a higher contamination risk to the surrounding environment (Aralu et al., 2025), whereas pollutants tend to be locally enriched during the dry season (Peng et al., 2025).

Spatial gradients in landfill leachate contamination levels also influences the variation patterns of soil microbial communities, resulting in significant spatial heterogeneity (Xu et al., 2024). The microenvironment created by valley topography affects soil physicochemical properties and heavy metal content, jointly contributing to the leachate-induced impact on the soil microbial community (Aralu et al., 2025).

Studies on landfill leachate predominantly focus on the impact of individual factors on bacterial communities, primarily including seasonal variations (Luo et al., 2025; Idbella et al., 2025; Akuaka et al., 2026), spatial distance (Xu et al., 2024), and soil depth (Hong et al., 2025), or pairwise interactions among these factors. However, the combined effects of season, distance, and soil depth remain underexplored (Lepcha et al., 2025; Zakaria et al., 2024). The effect of pH on bacterial community diversity and functionality (Kerfahi et al., 2024; Hong et al., 2025), as well as the combined influence of pH and heavy metals on bacterial community composition in polluted environments such as mining areas (Zhao et al., 2025), has been investigated. However, limited research has explored the intrinsic linkages between pH and Pb in co-regulating bacterial community composition and functionality. Investigation on spatiotemporal dynamics of bacterial community structure and function in valley-type landfills remains scarce (Xiang et al., 2024). This study bridges these gaps by elucidating the multifactorial coupling effects and pH-Pb co-regulation mechanisms. Our findings advancing microbial ecology in leachate-polluted zones, provide theoretical foundations for leachate-contaminated soil remediation, and reveal the role of valley-type landfills in global C/N/S cycle.

We conducted field sampling over a complete hydrological year at a valley landfill in Sichuan, Southwest China. The investigation revealed that leachate-impacted groundwater exhibited elevated levels of ammonia-nitrogen (NH_4_-N) and Pb, exceeding regulatory limits (Meng et al., 2024). Therefore, this study aims to elucidate the leachate’s effects on the soil environment. The study area was stratified into upstream (US), midstream (MS), and downstream (DS) zones based on potential leachate influence, with mountaintop and unaffected areas designated as controls (CK). Soil physicochemical properties and bacterial communities diversity were demonstrated (Louca et al., 2016). Our research examines how season, soil layer and topographic position affect the diversity, composition, and functioning of soil bacterial communities. Based on this, we sought to investigate: (1) How does leachate-induced variation in soil bacterial diversity respond to seasonal changes, and how do characteristic leachate pollutants mediate changes in community composition and C/N/S-cycling functions by modifying soil physicochemical properties? (2) How do microtopographic gradients within the valley regulate soil bacterial diversity, community composition, and functional attributes? These findings will advance understanding of how spatio-temporal interactions shape soil biodiversity conservation in leachate-affected systems, reveal microbial adaptation mechanisms, and inform seasonal management strategies for valley-type landfills.

## 2 Materials and methods

### 2.1 Site description

The study site is a large-scale operating municipal solid waste landfill in Xingwen County, Sichuan Province, Southwest China (28.28° N, 105.27°E, altitude 403 meters). The annual average temperature is 21 °C, ranging from 11 °C (January) to 32°C (July). The annual precipitation is about 1,300 mm, with approximately 30 mm in the dry season and 300 mm in the wet season. The landfill is located in the headwaters of a seasonal stream within a valley area. The regional stratum primarily consists of sandstone from the Xujiahe Formation of the Permian System (Meng et al., 2024). Pollutants can readily migrate along the valley and through geological strata, potentially expanding the pollution range. Therefore, these regional characteristics make this site ideal for examining landfill impacts on the surrounding soil environment, bacterial community structure, and how the composition and variation of bacterial functional groups are influenced by seasonal variations, topography, and distance gradients.

### 2.2 Sampling point layout and collection

Preliminary research indicated that the landfill leachate had elevated pH (alkaline), with concentrations of Pb, chemical oxygen demand (COD_Mn_), and NH_4_^+^-N exceeding environmental standards, particularly during the dry season (Meng et al., 2024). Accordingly, soil monitoring points were established at approximately equal intervals along the upstream-downstream gradient, targeting relatively flat terrain within the valley, with supplemental sampling at visible leachate seepage points. Sampling locations were georeferenced using GPS devices.

**FIGURE 1:**
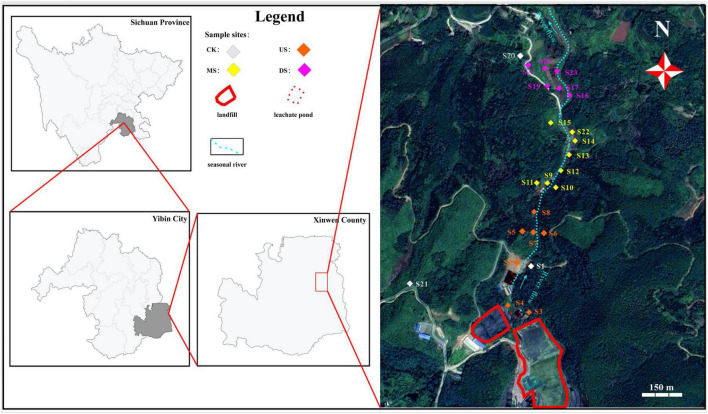
Landfill sampling sites of study area. CK (White color): soil samples far from the seasonal river and are less polluted by the landfill; US (orange color), MS (yellow color), DS (purple color) represent soil samples collected at the US, MS and DS of the seasonal river, respectively.

Soil samples were collected during three hydrological seasons: normal season (April 2023), wet season (September 2023), and dry season (January 2024). A total of 24 sampling points were established, with collections from both topsoil (0–10 cm) and subsoil (10–20 cm) horizons at each point, yielding 144 soil samples (Figure 1). Following removal of macrofauna and gravel, approximately 1 kg of soil was retained from each sample. Subsamples for high-throughput sequencing (approximately 30 g) were placed in sterile containers and maintained at low temperatures in coolers. Other subsamples were allocated to sampling bags for physicochemical analysis. All samples were promptly transported to the laboratory and stored at −80°C (for molecular analysis) and 4°C (for physicochemical analysis) until processing (Cai et al., 2024; Yang T. Y. et al., 2022)

### 2.3 Determination of soil physical and chemical factors in soil

pH was measured potentiometrically in a 1:2.5 (w/v) soil-water suspension (Kolodziej et al., 2024). Soil organic matter (SOM) was determined by the potassium dichromate oxidation method (external heating method) (Liu et al., 2022). Cation exchange capacity (CEC) was determined by the ammonium acetate exchange method (Bi et al., 2023). Total nitrogen (TN) was determined by the semi-micro Kjeldahl method (Nelson and Sommers, 1980). Total phosphorus (TP) and total potassium (TK) were determined by acid digestion followed by inductively coupled plasma optical emission spectrometry (ICP-OES) (Jin et al., 2023). Available potassium (AK) was determined using the ammonium acetate extraction-flame photometry method (Zhu et al., 2021). Available phosphorus (AP) was extracted with 0.5 M sodium bicarbonate (Olsen method) and determined by the molybdenum blue method. Alkaline hydrolysable nitrogen (AN) was determined by the alkaline hydrolysis diffusion method (Yang T. Y. et al., 2022). Heavy metals (Hg, As, Cr, Ni, Cu, Zn, Cd, and Pb) were quantified using acid digestion followed by inductively coupled plasma mass spectrometry (ICP-MS) (dos Santos et al., 2022).

### 2.4 Soil DNA extraction and microbial analysis

Soil microbial DNA was extracted using the E.Z.N.A.^®^ soil DNA kit (Omega Bio-tek, Norcross, GA, United States) according to the manufacturer’s instructions. Bacterial PCR amplification primers were 338F (5′-ACTCCTACGGGAGGCAGCAG-3′) and 806R (5′-GGACTACHVGGGTWTCTAAT-3′). After detection and recovery of the amplification products, library construction and high-throughput sequencing were completed by Shanghai Majorbio Bio-pharm Technology Co., Ltd., using the Illumina MiSeq sequencer.

Post-sequencing analysis was primarily performed on the Majorbio Cloud Platform.^1^ VSEARCH software was used to cluster sequences into operational taxonomic units (OTUs) at a 97% similarity threshold. Representative sequences of OTUs were classified taxonomically using the RDP classifier (Ribosomal Database Program) with the Bayesian algorithm, and community composition for each sample was statistically analyzed at different taxonomic levels. Although ASVs offer higher resolution, OTU-based analysis was chosen for its robustness in characterizing soil microbial community structure and its suitability for functional prediction in ecological studies. Alpha diversity (Chao1, Shannon) was calculated from OTU tables.

### 2.5 Statistical analysis

Soil physicochemical properties were processed in Excel and SPSS (descriptive statistics and significance tests). Figures were plotted in Origin 9.0. Bray-Curtis PCoA, and ANOSIM determined microbial community dissimilarities. Kruskal-Wallis tests identified taxonomic and functional group differences (FAPROTAX-predicted) across soil layers, seasons, and topographic positions. Pearson correlation assessed microbe-environment relationships. Co-occurrence networks were constructed in Python (NetworkX 1.11) using the top 100 OTUs (SparCC |*r*| > 0.5, *p* < 0.01) (Hagberg et al., 2008), with topological metrics calculated and visualized in Gephi 0.10.1 (Bastian et al., 2009). Partial least squares path modeling (PLS-PM) was implemented in R (v4.5.1, plspm v0.4.9). Random forest (RF) analysis quantified the relative importance of season, soil depth, and landfill distance in shaping soil physicochemistry, bacterial community traits (dominant genera, α-diversity, network structure), and predicted C/N/S cycling functions. Three-way ANOVA was also performed using SPSS 19.0 software (SPSS Inc., Chicago, IL, United States) to examine the interactive effects of pollution source distance, soil horizon, and seasonal variation on soil physicochemical properties and microbial community composition.

## 3 Results

### 3.1 Soil bacterial community profiles under leachate influence

**FIGURE 2:**
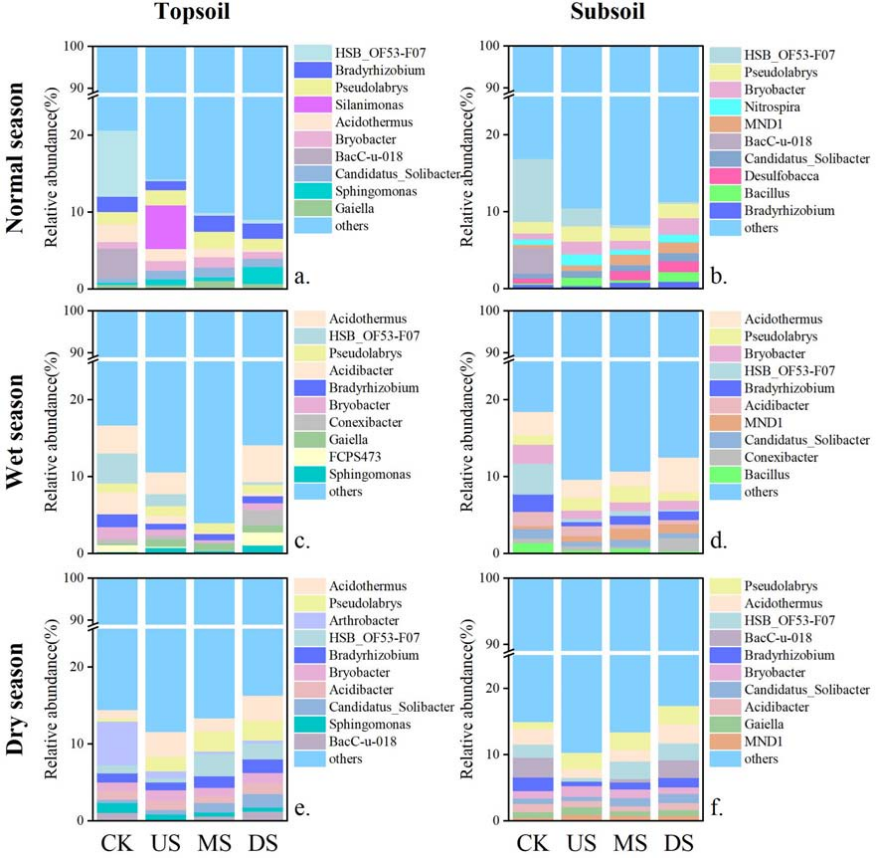
Distribution of soil bacterial communities at the genus level in topsoil and subsoil layers during different periods. Percentage bar chart of dominant microorganisms at the genus level for each sample point. **(a–f)** The top 10 most abundant genera identified within that specific sample group (topsoil/subsoil, normal/wet/dry season). OTUs not identified to genus are merged into “Others.” The bar chart shows the proportion of dominant genus abundance at each sample point. CK: soil samples far from the seasonal river and are less polluted by the landfill; US MS, DS represent soil samples collected at the upstream, middle stream and downstream of the seasonal river, respectively.

Leachate significantly increased soil bacterial diversity (Chao1 and Shannon indices), especially at sites near the leachate pond (MS) (Supplementary Figure S1). *Proteobacteria, Chloroflexi*, and *Acidobacteriota* were the dominant phyla (relative abundance > 10%) across all seasons, while *Firmicutes, Nitrospirota, Verrucomicrobiota*, and *Actinobacteriota* peaked in the dry season (Supplementary Figures S1, S2c–f). *Proteobacteria* and *Acidobacteriota* were most abundant at US sites. In contrast, leachate exposure reduced *Chloroflexi* abundance by over 50%, with the maximum reduction occurring in the normal season (Supplementary Figure S3). At the genus level, *Pseudolabrys* remained dominant (Figure 2). The relative abundance of *Pseudolabrys* was 2–5 times higher than that in CK during the dry season. Conversely, the leachate-sensitive genus *Bryobacter* exhibited lower abundances in the wet season.

### 3.2 Correlation between soil bacterial community, C/N/S-cycling functional groups and soil physicochemical properties

**FIGURE 3:**
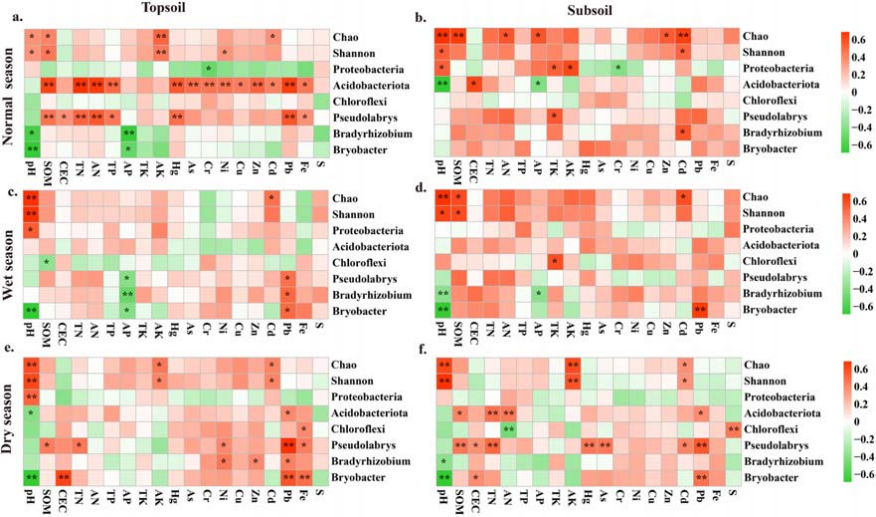
Effects of environmental factors on bacterial communities during the normal season **(a,b)**, wet season **(c,d)**, and dry season **(e,f)** (Heatmaps showing the correlations between the Chao index, Shannon index, dominant bacteria, and environmental factors. The intensity of the color indicates the strength of the correlation. The legend on the right represents the correlation coefficient: colors closer to the upper part of the legend indicate a stronger positive correlation, and vice versa for a stronger negative correlation. “*” denotes significance, and “**” denotes extreme significance.

**FIGURE 4:**
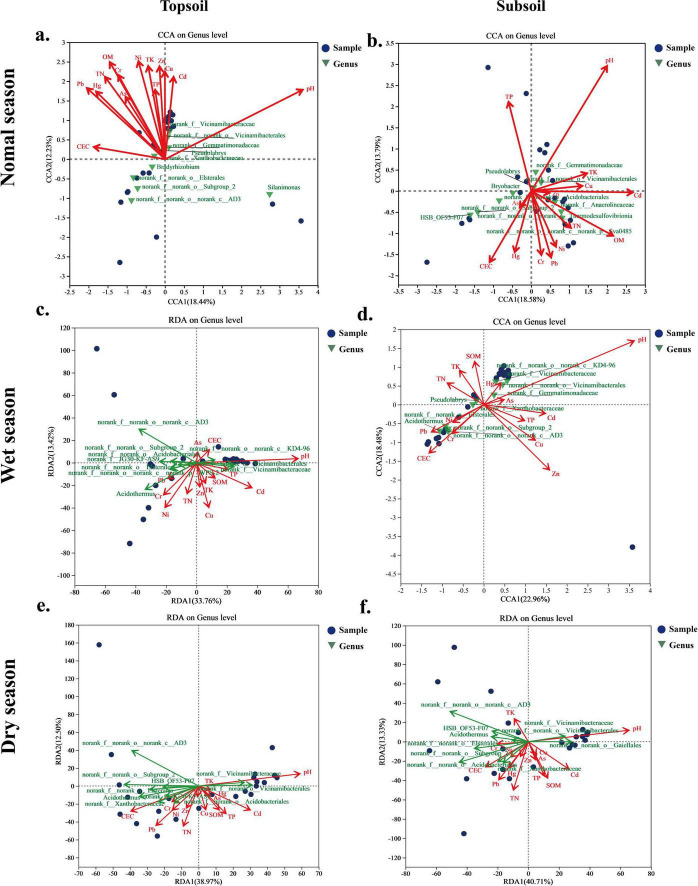
Effects of environmental factors on bacterial communities during the normal season **(a,b)**, wet season **(c,d)**, and dry season **(e,f).**

As presented in Figure 3, soil bacterial diversity (Chao, Shannon) showed a significant positive correlation with pH (*P*< 0.05). *Bryobacter* exhibited a significant positive correlation with Pb (*P*< 0.05) in all samples except subsoil during the normal season. In topsoil, *Pseudolabrys* was significantly positively correlated with Pb (*P* < 0.05), while *Bryobacter* was significantly negatively correlated with pH (*P* < 0.05). Soil pH was consistently identified as a key factor shaping soil bacterial communities across all seasons (Figure 4). During the normal and wet seasons, both pH and SOM were the dominant influencing factors. In the dry season, Pb, Cd, and Zn emerged significant factors influencing bacterial communities.

**FIGURE 5:**
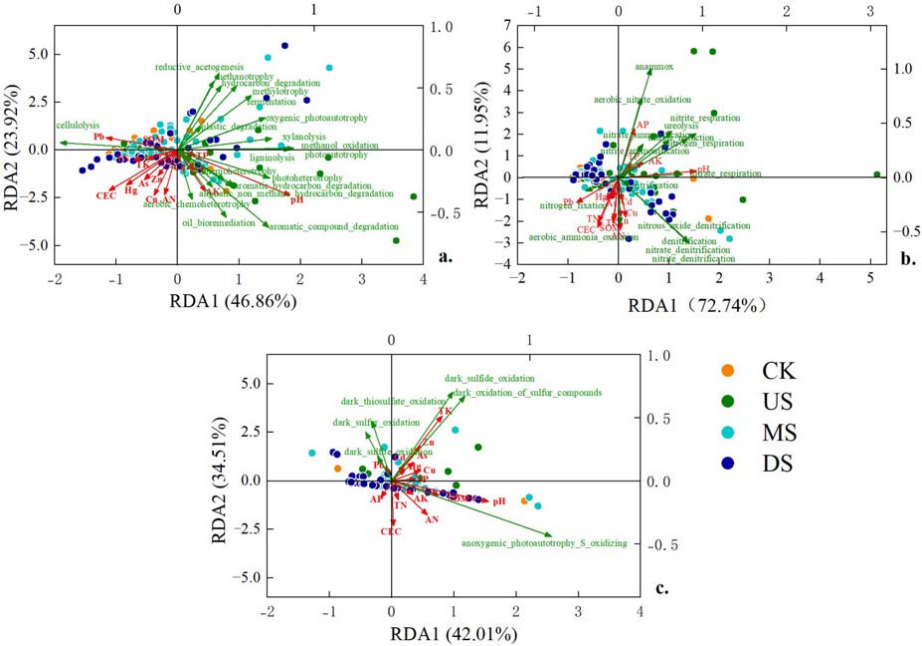
Effects of environmental factors on soil microbial C-cycling functional groups **(a)**, N-cycling functional groups **(b)**, and S-cycling functional groups **(c)** in different regions. CK: soil samples far from the seasonal river and are less polluted by the landfill; US MS, DS represent soil samples collected at the upstream, middle stream and downstream of the seasonal river, respectively.

The relative abundance of C-cycling functions was significantly positively correlated with pH (*P* < 0.001), while negatively correlated with heavy metals (Pb, *P* < 0.001; Hg, *P* = 0.006; As, *P* = 0.038) and soil nutrient ions (TK, CEC, *P* < 0.001). pH was the predominant contributor, explaining 27% of the variance in C-cycling functions (Figure 5), followed by Pb and CEC (11%). Similarly, pH, Pb, and CEC were key drivers of N-cycling functions, accounting for 45, 14, and 5.6% of variance, respectively. Among nutrient indicators, TN (*P* = 0.02), AN (*P* = 0.04), and AP (*P* = 0.05) exhibited significant positive correlations with the abundance of most N-cycling functions. The relative abundance of S-cycling functions was significantly positively correlated with pH (*P* < 0.001) and TK (*P* = 0.006). A negative correlation with CEC was observed but was not significant (*P* = 0.25). Consequently, the main factors influencing the S-cycling functions were pH and TK, contributing 24 and 17% to the explained variance, respectively.

### 3.3 Soil bacterial community network co-occurrence and predicted functions

**FIGURE 6:**
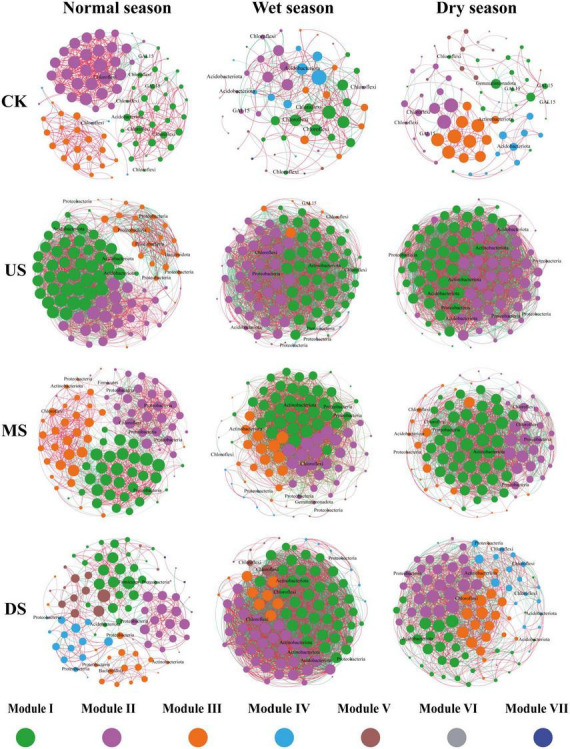
Soil microbial network structure diagram. (Based on the top 10 dominant OTU by abundance, absolute correlation coefficient value greater than 0.5, *p*-value less than 0.01. Nodes represent different OTU, node size represents degree, different colors represent different modules, lines between nodes represent associations, red represents positive correlation, green represents negative correlation, and node labels are the phyla of the top 10 abundant OTU.) CK: soil samples far from the seasonal river and are less polluted by the landfill; US MS, DS represent soil samples collected at the upstream, middle stream and downstream of the seasonal river, respectively.

Based on calculations of bacterial network topological characteristics (Figure 6 and Supplementary Table S1), nodes in these grouped networks were assigned to up to 7 modules. Higher node number, link number and average degree were observed at US and MS zones, which were directly affected by leachate. Network connectivity and clustering coefficient were enhanced by leachate, whereas modularity was reduced. Network structure was more integrated and stable. The shortened average path length suggested enhanced efficiency of information transmission and material exchange within the network.

Analysis of correlation patterns within the networks revealed that the reduction in the proportion of positive correlations indicated a shift in microbial interactions from predominantly cooperative associations toward more complex networks involving both positive and negative correlations. Leachate effects on bacterial co-occurrence network were more pronounced in wet season, whereas microbial communities influenced by leachate exhibited greater network stability in the dry season.

**FIGURE 7:**
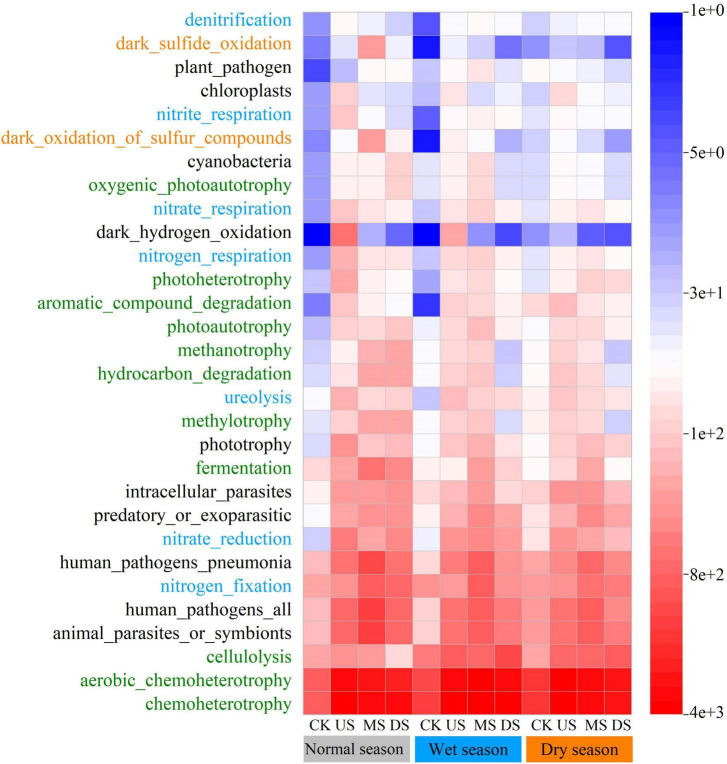
Seasonal and regional variation in the top 30 dominant soil bacterial functions, annotated by the FAPROTAX database. (Seasons: Normal, Wet, Dry. CK: soil samples far from the seasonal river and are less polluted by the landfill; US MS, DS represent soil samples collected at the upstream, middle stream and downstream of the seasonal river, respectively. Functional labels on the left are color-coded: C cycle (green), N cycle (blue), S cycle (orange), other functions (black).)

In this study, a total of 72 predicted functional categories were annotated based on the FAPROTAX database, and the top 30 dominant functional categories of soil bacteria were presented in Figure 7. The predicted functional composition was: C cycle (26.4%), N cycle (22.2%), S cycle (8.3%), and other functions (including animal parasites or symbionts, human pathogens all, human pathogens pneumonia, etc., 43.1%). During the wet and normal seasons, sites directly exposed to leachate had higher abundances of C/N/S-cycling functions. In contrast, during the dry season, leachate-impacted sites exhibited the lowest abundances.

### 3.4 Interaction of bacterial communities, network structures, predicted functions, with environmental factors

**FIGURE 8:**
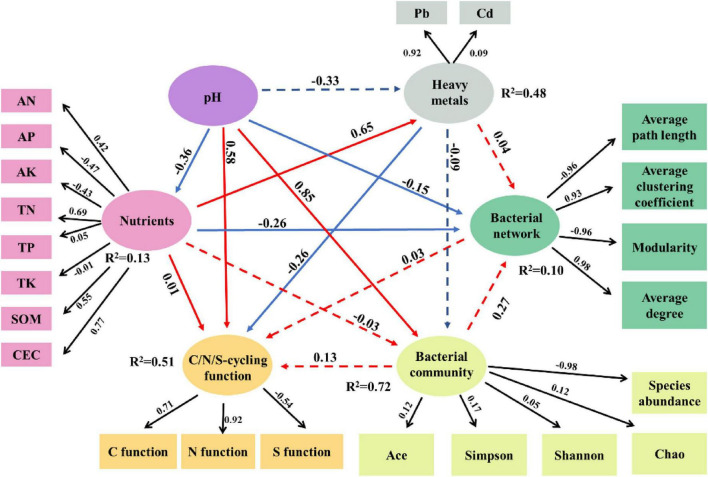
PLS-PM results (Goodness = 0.39, *n* = 144). Arrow annotations show path coefficients (solid = significant at *p* < 0.05; dashed = non-significant). Color-coded arrows: blue = negative, red = positive effects. Arrow labels indicate latent-observed variable loadings.

The PLS-PM results (Goodness of Fit = 0.39) demonstrated that the conceptual model explained 72% of the variation in bacterial community, 51% of the predicted C/N/S-cycling functions, and 10% of the bacterial networks (Figure 8). Soil pH exerted the most substantial influence on microbial properties, including C/N/S-cycling functions, bacterial community structure, and network stability (Figure 8), followed by soil heavy metals and nutrient indicators. Soil pH showed a negative correlation with soil nutrients (−0.36), but positive correlations with soil C/N/S-cycling functions (0.58), soil bacterial network structure (0.33), and bacterial community (0.85). Soil nutrients had positive effects on heavy metals (0.65) and C/N/S-cycling functions (0.01), but negative effect on bacterial network (−0.26), primarily through TN, CEC, and SOM. Heavy metals were negatively correlated with soil C/N/S-cycling functions (−0.26), with Pb exhibiting a high loading of 0.92.

In summary, soil microbial composition in the study area was primarily governed by soil pH, followed by soil heavy metals (particularly Pb) and nutrients (SOM, CEC, and TN). These factors collectively shaped bacterial community structure, network complexity, and C/N/S-cycling functions.

## 4 Discussion

### 4.1 Impact of landfill leachate contamination on soil bacterial composition

Landfill leachate alters soil bacterial communities and their diversity by modifying soil physicochemical properties and heavy metal concentrations (Naeem et al., 2025). High-throughput sequencing-based studies revealed significantly higher α-diversity (Chao1 and Shannon indices) at leachate-impacted sites. The dominant bacterial phyla were *Proteobacteria, Chloroflexi*, and *Acidobacteriota*, consistent with previous studies (Cai et al., 2024; Zakaria et al., 2024). *Proteobacteria* and *Acidobacteriota* demonstrated heavy metal tolerance, achieving maximal relative abundances at leachate-adjacent US sites (exceeding controls), consistent with previous studies (Lepcha et al., 2025). In contrast, the sensitive *Chloroflexi* peaked in CK and declined toward the leachate source (most notably at US sites). The relative abundance of *Chloroflexi* under leachate-induced stress (Zabihollahi et al., 2024), was likely due to the high concentrations of COD_Mn_, NH_4_^+^-N, salt ions, and Pb in the leachate. These pollutants are known to inhibit *Chloroflexi*’s metabolic activity, disrupt cell membrane integrity, and lead to cell death.

*Pseudolabrys* exhibited minimal seasonal variation compared to other genera, which showed substantial fluctuations across seasons. *Pseudolabrys* and *Bradyrhizobium* accumulated at leachate-impacted sites (adapting to Pb, CODMn, ammonium, and salts). *Pseudolabrys* possesses inherent resistance mechanisms against heavy metal stress (Xu M. Y. et al., 2025), partly attributed to its ability to mitigate harmful substances via adsorption, absorption, transformation, and efflux (Hu and Yang, 2025). Additionally, *Pseudolabrys* can alleviate the inhibitory effects of heavy metals by promoting plant uptake of heavy metals from the soil and secreting siderophore chelates (Kushwaha et al., 2023; Xu L. et al., 2025). However, abundance of *Pseudolabrys* may decline above a toxicity threshold, while taxa with superior metal resistance (e.g., Gaiella, Haliangium) potentially increase (Wu et al., 2024). Although *Bradyrhizobium* demonstrates sensitivity to various factors, including soil pH, heavy metal content, and moisture content (Barquero et al., 2017), it exhibits strong resilience through metabolic versatility, stress resistance, and diverse carbon substrate utilization capabilities. Physiologically, *Bradyrhizobium* maintains osmotic balance via compatible solute production (e.g., glycine betaine), while molecularly, it activates antioxidant enzyme systems such as superoxide dismutase and catalase (Sharma et al., 2025). Its heavy metal resistance mechanisms (Montes-Montes et al., 2025) include enhanced efflux pump and transporter activity for metal ion removal (Lamin et al., 2021), production of metal-reducing enzymes (Arregui et al., 2021), and stress-induced radical-mediated metal detoxification (Vezza et al., 2022).

*Bryobacter* abundance decreased in leachate-impacted soils, despite its known tolerance to salt and heavy metals (Liu et al., 2025) and role in biogeochemical cycling (He W. J.et al., 2023; Wang et al., 2024). These results suggest that leachate pollution exceeded its adaptive threshold or favored more competitive taxa.

### 4.2 Impact of landfill leachate contamination on soil bacterial co-occurrence networks

Microbial co-occurrence networks analysis enables identification of potential microbial interactions, phylogenetic relationships, functional associations, and niche partitioning (Li D. et al., 2024; Li J. Y. et al., 2024; Yin et al., 2024). In these networks, nodes represent microbial taxa and edges denote significant correlations between microbial taxa (Deng et al., 2024). The dominant phyla *Chloroflexi, Acidobacteriota, Proteobacteria*, and *Actinobacteriota* functioned as keystone taxa maintaining network stability under leachate influence, consistent with previous findings (Yin et al., 2024). *Proteobacteria* play a key role in maintaining soil bacterial network stability (Li D. et al., 2024). Under severe co-pollution stress, surface microbial communities underwent distinct co-occurrence pattern shifts. Specifically, dominant taxa enhanced their adaptability, resistance, and competitiveness to adverse conditions by strengthening interactions (Li D. et al., 2024). Synergistic interactions outnumbered antagonistic ones in leachate-affected communities (Yin et al., 2024). Toxic constituents like Pb presumably eliminated sensitive bacteria, leading to functional simplification, reduced microbial diversity, and a shift in community composition toward tolerant and metabolically versatile taxa. This simplification was reflected in network structure, with less modules detected at upstream sites during the normal (three) and dry seasons (two) (Li M. et al., 2025).

Network complexity enhanced during the wet season, aligning with Chen et al. (2021). Elevated humidity and runoff input during the wet season likely promote the diffusion of carbon and nitrogen resources, increasing bacterial diversity and enhancing both cooperative and competitive interactions between (Sun et al., 2017).

Under the stress of trace pollutants such as heavy metals from leachate, microorganisms resist environmental disturbances by developing specialized symbioses and enhancing stable network architectures characterized by higher modularity and positive links (Siles et al., 2021). This may explain the higher modularity observed during the dry and normal seasons.

The network in the normal season displayed higher modularity and average path length, with the highest percentage of positive links, contrasting with wet season minima. The relatively stable soil environment and resource availability during the normal season may promote microbial differentiation into discrete functional modules, thereby reducing inter-module competition (Qin et al., 2023).

### 4.3 Impact of landfill leachate contamination on C/N/S cycle functions of soil bacteria

Understanding the C/N/S cycling dynamics in landfill-affected ecosystems represents a focal area in soil ecology and biogeochemistry (Li D. et al., 2024; Liu et al., 2025). Leachate alters soil microbial community diversity, which in turn influences soil C/N/S cycle functions, thereby underscoring the necessity to elucidate microbial community structure and function (Lepcha et al., 2025).

Functional assignments derived from FAPROTAX represent predicted ecological potential functions, rather than direct evidence of gene presence or metabolic activity, as recommended in previous relevant studies (Hu et al., 2025; Li Y. et al., 2025). Accordingly, these results only indicated putative microbial functional profiles and should not be overinterpreted as definitive functional activities. The abundances of these C/N/S-cycling functional groups were highly sensitive to soil physicochemical properties (Gu et al., 2022; Yang Z. C. et al., 2022). The soil pH in this study varied widely and significantly influenced the abundances of these functions (Lauber et al., 2009; Griffiths et al., 2011). This substantial pH variation could be attributed to the input of alkaline landfill leachate (Lemanowicz et al., 2023).

Our research revealed a positive correlation between soil pH and the abundance of most C/N/S-cycling functions. In contrast, soil heavy metal composition generally exerted negative effects on these functions. Consistent with existing studies, heavy metal pollution can significantly impair microbially mediated ecological functions (Ke et al., 2023). Most heavy metals measured in this study were inversely correlated with the abundance of these functions. Among them, Pb exhibited the most pronounced inhibitory effect on the abundance of C/N/S cycling functions, followed by Hg, As, Cu, and other heavy metals (Chen et al., 2024; Jiang et al., 2025).

Notably, cellulolysis, ranked among the top three most abundant C-cycling functions, constituted an exception. It appeared tolerant to certain heavy metals but was disfavored in alkaline conditions. Beyond soil pH and heavy metals, other physicochemical properties, such as TK content, had a significant impact on the abundance of specific S-cycling functions, particularly those for dark sulfide oxidation and dark oxidation of sulfur compounds. Gu et al. (2022); Li D. et al. (2024) have found that landfill leachate leakage introduces S-containing substances into the soil, leading to a substantial increase in the abundance of S-cycling functions. The complex salt composition of leachate, particularly inputs of K-SO4^2+^ salts, may be a key factor stimulating S-cycling functions.

### 4.4 Multi-factor interactions of bacterial communities, network structure, and C/N/S-cycling functions

Distance was the core driving factor, with importance significantly surpassing that of season and soil layer (Supplementary Figure S4 and Supplementary Table S2). An interactive effect between distance and season was observed. Sites directly affected by leachate exhibited greater microbial diversity and C/N/S-cycling functions. Soil pH was a key determinant of microbial community diversity across seasons and soil layers (Park et al., 2025), with significant negative correlations (*P* < 0.05) between pH and the relative abundances of *Bradyrhizobium* and *Bryobacter*, indicating their preference for neutral to slightly acidic conditions (Li J. Y. et al., 2024; Shu et al., 2023; Li Y. et al., 2025). Excessively alkaline conditions (pH > 8.0) reduced the solubility of essential nutrients (e.g., phosphorus, iron), thereby impairing *Bradyrhizobium* metabolism and nitrogen fixation efficiency. Similarly, elevated pH (> 7.5) can alter habitat chemistry for *Bryobacter*, reducing carbon and nitrogen availability and ultimately diminishing its biomass and ecological function. Our PLS-PM model indicated that soil pH directly influenced C/N/S-cycling functions, microbial network structures, and microbial community characteristics. Additionally, soil pH negatively affected soil nutrients, which were positively associated with soil heavy metals, ultimately exerting an indirect negative impact on C/N/S-cycling functions through heavy metals.

Pb had a secondary effect on bacterial communities, but negatively affected C/N/S-cycling functions (Chen et al., 2024; Jiang et al., 2025). Pb weakly altered bacterial network structure, and indirectly affected C/N/S cycling functions via bacterial community modulation, reducing carbon cycle enzyme activity and altering related functional groups/OTUs (Li et al., 2018; Wang et al., 2022). Pb indirectly disrupted S cycling by suppressing sulfur-oxidizing taxa like *Iobacillus*. Qin et al. (2023) found that Pb-tolerant bacterial groups such as *Actinobacteriota* increased in abundance with Pb levels. These groups can replace microbial taxa inhibited by Pb, thereby maintaining the stability of community diversity. Bacterial groups maintain network connections by retaining core functional taxa (Golebiewski et al., 2014).

**FIGURE 9:**
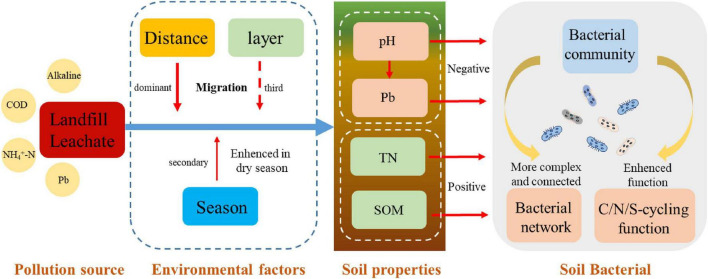
Multi-factor regulation of landfill leachate on soil properties and microbial responses in valley ecosystems.

Excessive COD and NH_4_-N in leachate altered soil nutrient composition. Soil TN, CEC, and SOM modified heavy metal composition, shaped bacterial communities and C/N/S cycling functions (Figure 9). Soil nutrients were poor in our study, but leachate infiltration altered pH, nutrient and heavy metal composition, due to its complex inorganic and organic constituents. No correlation between soil nutrient deficiency and C/N/S-cycling functions (Wang et al., 2023); Yang Y. et al. (2022).

Based on the above findings, we conclude that landfills have a significant impact on the microbial community structure, diversity, and C/N/S-cycling functions in the study area, primarily mediated directly and indirectly by the characteristic high pH and Pb content of the leachate. This mechanistic understanding helps target risk management against the most impactful pollution factors in landfills, thereby mitigating risks of ecological imbalance from leachate leakage.

## 5 Conclusion

This study investigated the response of soil bacterial community structure and function to landfill leachate contamination. A significant increase in bacterial community diversity at the directly affected sites. The results demonstrated that variations in leachate concentration leading to a significant increase in the relative abundance of *Proteobacteria* and *Pseudolabry*, nevertheless reducing the relative abundance of *Chloroflexi*. Soil pH and Pb were the predominant drivers of microbial community structure, diversity and C/N/S-cycling functions. The regulatory effects of season, distance and soil layer on soil abiotic and biotic properties exhibited significant differences, with distance being the core driving factor. Additionally, an interactive effect was observed between distance and season. The promoting effect of distance on microbial diversity was more pronounced during the wet season. The findings provided a scientific basis for understanding the environmental response mechanisms of soil ecosystems surrounding valley-type landfill sites.

Future laboratory simulations should incorporate metagenomic sequencing and enzyme activity assays under varying leachate concentrations to demonstrate the interactive effects of key contributing factors. This investigation will help reveal the mechanistic impacts of leachate on soil microbial communities and their contributions to C/N/S cycle.

## Data Availability

The raw sequencing data have been deposited in the NCBI SRA database under BioProject accession number PRJNA1434660, which is accessible at https://www.ncbi.nlm.nih.gov/bioproject/PRJNA1434660.

## Footnotes

1
https://cloud.majorbio.com
