# Supplementary material for: pH and Pb co-regulate soil bacterial communities and C/N/S Cycling processes in valley-type landfills under multi-factor interactions
Source: Front Microbiol. 2026 Mar 24;17:1780940. doi: 10.3389/fmicb.2026.1780940 (PMC13055613; doi:10.3389/fmicb.2026.1780940)
Supplement: Supplementary file 1 [file Data_Sheet_1.docx]

**Supplementary Tables**

Table S1. Topological properties of the co-occurrence networks of the bacterial community in soils.

|  | Normal | | | |  | Wet | | | |  | Dry | | | |
| --- | --- | --- | --- | --- | --- | --- | --- | --- | --- | --- | --- | --- | --- | --- |
| Network Parameters | CK | US | MS | DS |  | CK | US | MS | DS |  | CK | US | MS | DS |
| Nodes | 88 | 98 | 96 | 96 |  | 59 | 99 | 99 | 98 |  | 72 | 96 | 99 | 98 |
| Links | 520 | 1820 | 1054 | 426 |  | 232 | 2590 | 1997 | 2922 |  | 172 | 2812 | 1682 | 1470 |
| Average degree | 11.82 | 37.14 | 21.96 | 8.88 |  | 7.86 | 52.32 | 40.34 | 59.63 |  | 4.78 | 58.58 | 33.98 | 30.00 |
| Network diameter | 6 | 4 | 6 | 8 |  | 7 | 7 | 6 | 5 |  | 12 | 4 | 6 | 4 |
| Modularity | 0.57 | 0.26 | 0.45 | 0.59 |  | 0.34 | 0.11 | 0.10 | 0.07 |  | 0.65 | 0.07 | 0.17 | 0.23 |
| Average clustering coefficient | 0.60 | 0.78 | 0.67 | 0.53 |  | 0.57 | 0.84 | 0.73 | 0.86 |  | 0.65 | 0.85 | 0.71 | 0.67 |
| Average path length | 2.68 | 1.87 | 2.12 | 3.02 |  | 2.45 | 1.75 | 1.89 | 1.48 |  | 3.63 | 1.43 | 1.92 | 1.94 |
| Percentage of positive links （%） | 93.85 | 52.8 | 66.98 | 74.88 |  | 67.67 | 50.12 | 50.03 | 51.30 |  | 87.79 | 50.25 | 50.71 | 52.79 |

Table S2. Results of P-values from Three-way ANOVA(season, layer, distance) for soil pH value, Pb content and dominated predicted C/N/S-clcyling function.

| Parameter |  | pH | Pb | CEC | TN | Bryobacter | Bradyrhizobium | Pseudolabrys |
| --- | --- | --- | --- | --- | --- | --- | --- | --- |
| Distance |  | 0.001* | 0.016 * | 0.020* | 0.001* | 0.947 | 0.002* | 0.025 * |
| Season |  | 0.392 | 0.743 | 0.116 | 0.577 | 0.999 | 0.939 | 0.044* |
| Layer |  | 0.995 | 0.928 | 0.264 | 0.056 | 0.216 | 0.023* | 0.381 |
| Distance× Season |  | 0.101 | 0.329 | 0.035* | 0.313 | 0.557 | 0.583 | 0.201 |
| Distance × Layer |  | 0.933 | 0.999 | 0.944 | 0.809 | 0.925 | 0.390 | 0.913 |
| Season × Layer |  | 0.767 | 0.926 | 0.960 | 0.307 | 0.727 | 0.026* | 0.699 |
| Distance× Season × Layer |  | 0.941 | 0.989 | 0.936 | 0.979 | 0.795 | 0.751 | 0.971 |
|  |  | C1 | C2 | C3 | N1 | N2 | N3 | S1 |
| Distance |  | 0.014* | 0.380 | 0.031* | 0.000* | 0.099 | 0.066 | 0.454 |
| Season |  | 0.019* | 0.012* | 0.199 | 0.326 | 0.609 | 0.700 | 0.475 |
| Layer |  | 0.000* | 0.479 | 0.002 | 0.347 | 0.035* | 0.068 | 0.12307 |
| Distance× Season |  | 0.240 | 0.775 | 0.264 | 0.011* | 0.842 | 0.502 | 0.274 |
| Distance × Layer |  | 0.353 | 0.899 | 0.486 | 0.716 | 0.582 | 0.952 | 0.380 |
| Season × Layer |  | 0.019* | 0.963 | 0.063 | 0.260 | 0.308 | 0.251 | 0.439 |
| Distance× Season × Layer |  | 0.665 | 0.996 | 0.695 | 0.931 | 0.670 | 0.681 | 0.680 |

Note: Seasons include: normal season, wet season, dry season. The soil layers include: upper layer and lower layer. distance include: CK, US, MS, DS.

* : Significance P value < 0.05.

C1: aerobic chemoheterotrophy, C2：cellulolysis, C3: chemoheterotrophy, N1: nitrogen fixation, N2: nitrate reduction, N3: ureolysis, S1：anoxygenic photoautotrophy S oxidizing.

**Supplementary Figures**


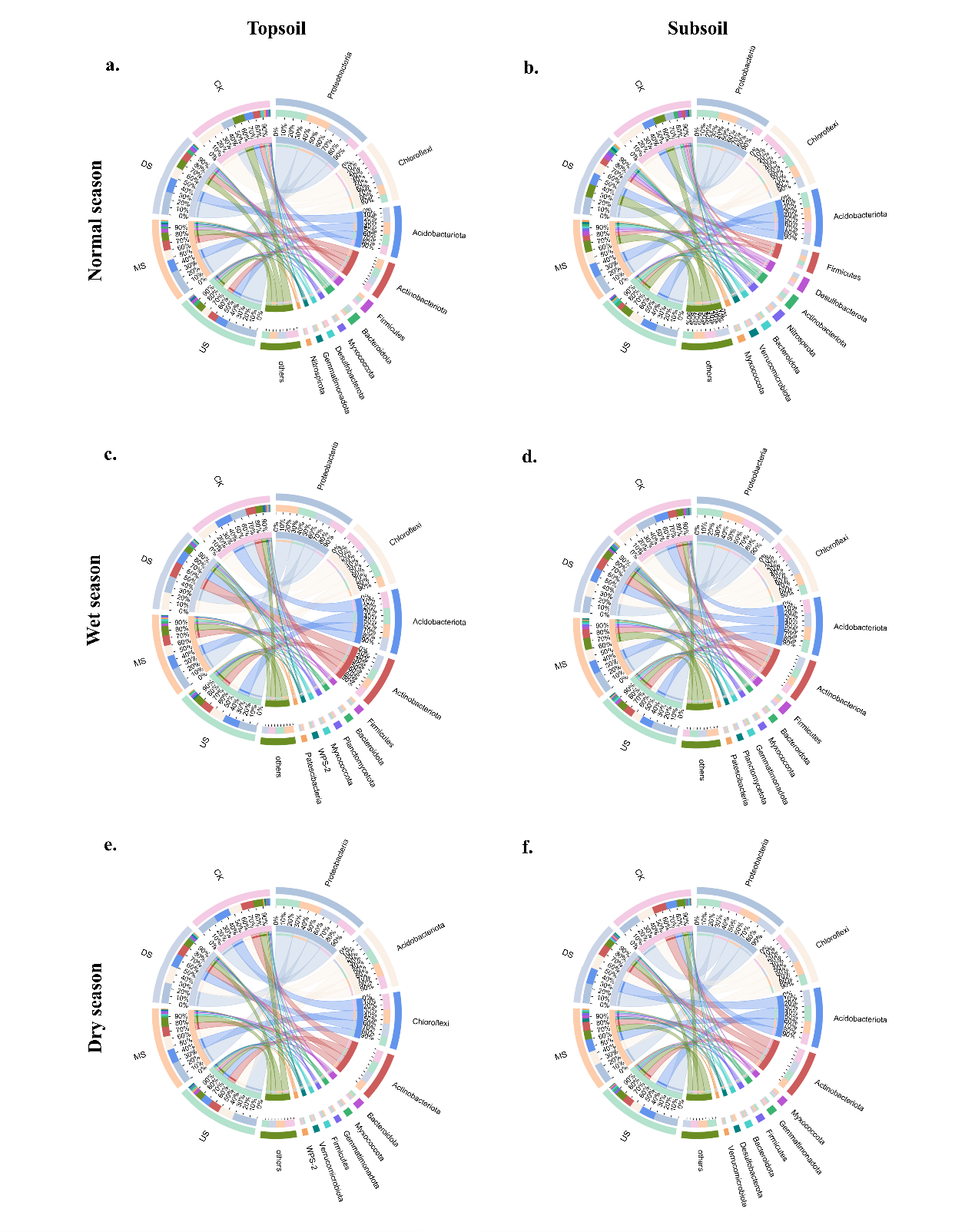


Fig. S1 Distribution of soil bacterial communities at the phylum level in topsoil (a, c, e) and subsoil (b, d, f) layers during different periods. (Circos sample-species relationship diagrams are typically used to display the distribution of microbial species present in different microbial samples. One side of the circle represents samples, the other side represents major dominant species, and the inner colored ribbons show the abundance distribution of different species in the samples.)


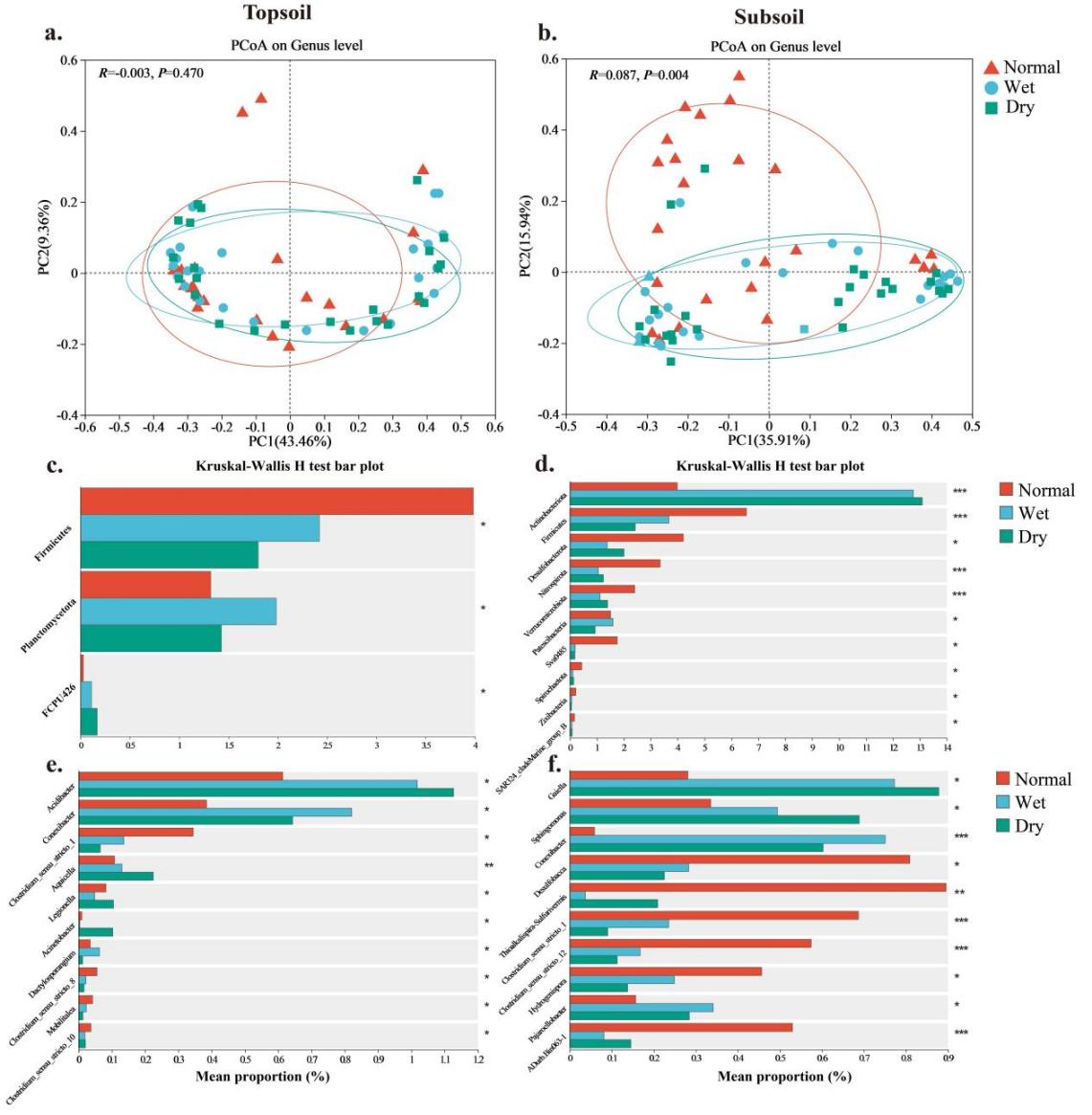


Fig. S2 PCoA analysis of bacterial communities in topsoil and subsoil soil during different periods (a, b), and changes in the relative abundance of fungal communities at the phylum level (c, d) and genus level (e, f)(Normal = Normal season; Wet = Wet season; Dry = Dry season; * P < 0.05; ** P < 0.01.)


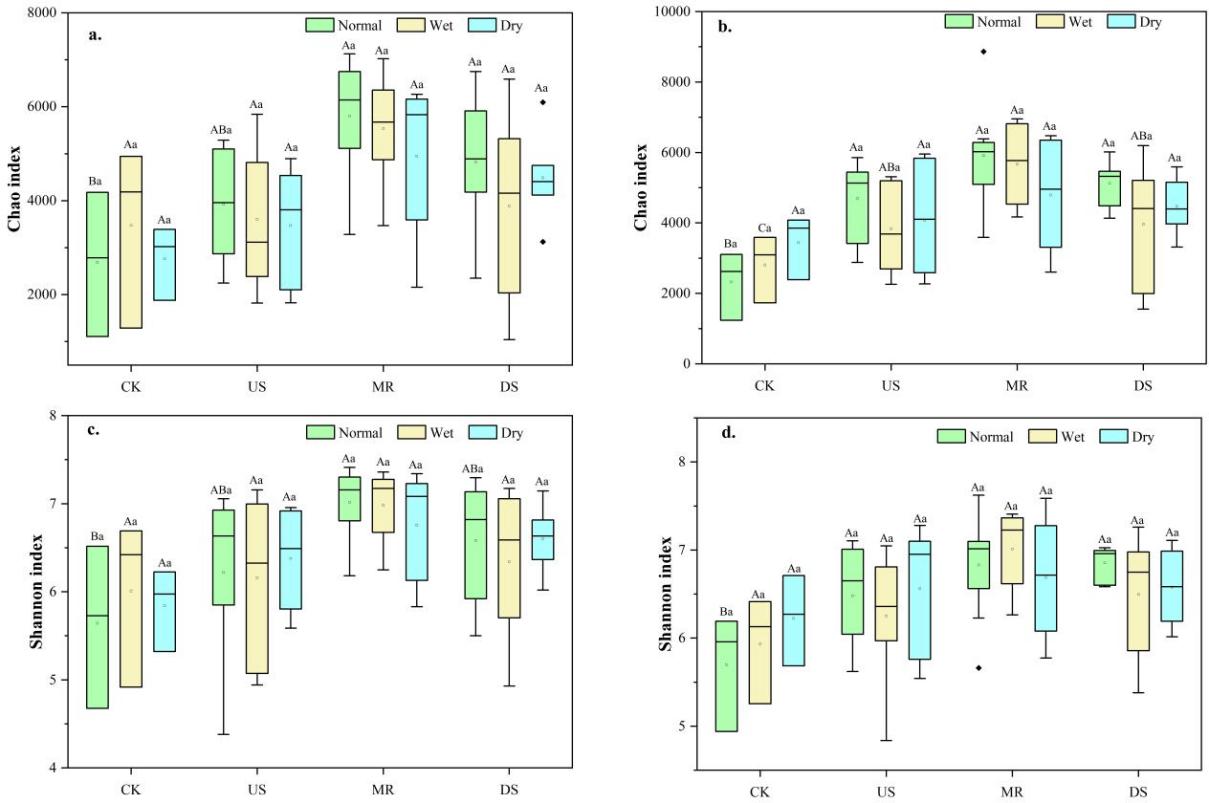


Fig. S3 Differences in soil bacterial α-diversity indices between topsoil (a, c) and subsoil soil (b, d) across different seasons(Normal = Normal season; Wet = Wet season; Dry = Dry season; CK = Control group; US = Upper Reach of the valley; MR = Middle Reach of the valley; DS = Downstream of the valley. Capital letters indicate significant differences in α-diversity indices among different topographic positions within the same soil layer and season; lowercase letters indicate significant differences in α-diversity indices among different seasons within the same soil layer and topographic position.)


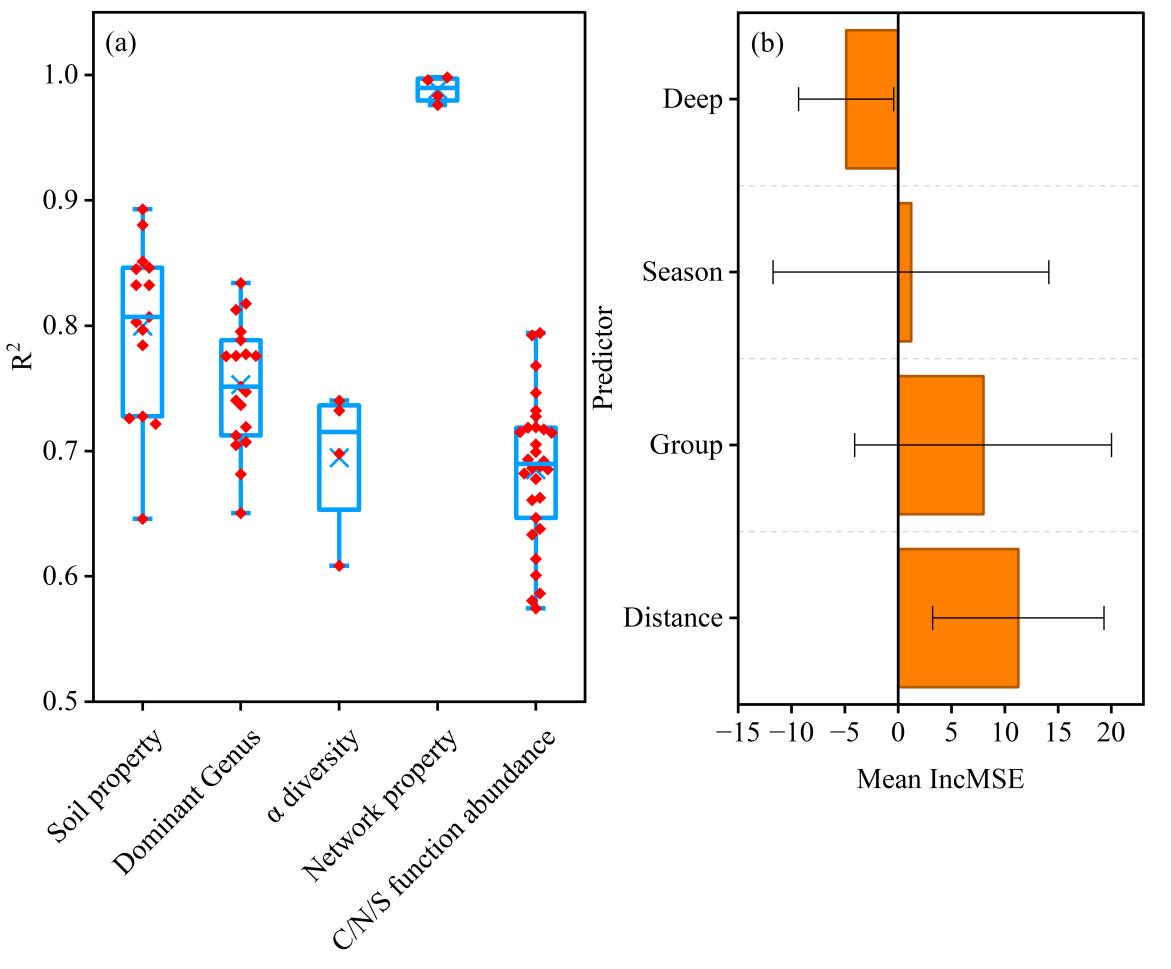


Fig.S4 Results of random forest model analysis (a: Reliability analysis of the random forest model; b: Importance analysis of season, soil layer, and distance）. Random forest models were constructed to disentangle the effects of spatial distance, season, and soil depth on soil physicochemical properties (pH, nutrient indicators, heavy metals), dominant bacterial genera, microbial diversity indices, network topological properties, and C/N/S-cycling functional groups. The models showed an average coefficient of determination (R²) of 0.74, indicating reliable predictive performance (Fig. S4a). Based on the mean increase in mean squared error (mean IncMSE), spatial distance (mean IncMSE = 11.3) was identified as the most critical factor, with the highest frequency (68 times) of being the best-ranked predictor. In comparison, group (mean IncMSE = 8.0, 3 times), season (mean IncMSE = 1.2, 4 times), and soil depth (mean IncMSE = -4.9, 1 time) had substantially lower importance (Fig. S4b, Table S2). These results collectively highlight that spatial distance is the primary driver governing the composition of both soil physicochemical and microbial communities in the study area.
